# The Protective Mechanism of Continuous Theta Burst Stimulation in the Acute Phase of Stroke Through Modulation of the Calcineurin/AKT/FOXO1 Signaling Pathway

**DOI:** 10.1002/cns.71017

**Published:** 2026-07-08

**Authors:** Pengkun Yang, Haocheng Qin, Bao Zhou, Shenyi Kuang, Meixi Liu, Yi Wu, Peng Yuan, Kewei Yu

**Affiliations:** ^1^ Department of Rehabilitation Medicine, Huashan Hospital Fudan University Shanghai China; ^2^ Department of Neurology, Huashan Hospital Fudan University Shanghai China; ^3^ National Center for Neurological Disorders Shanghai China; ^4^ Institute for Translational Brain Research Fudan University Shanghai China

**Keywords:** acute phase, Akt/FOXO1 signaling, continuous theta burst stimulation, extrinsic apoptosis, ischemic stroke

## Abstract

**Aims:**

To investigate the neuroprotective effects of continuous theta‐burst stimulation (cTBS) during the acute phase of ischemic stroke and its impact on neuronal apoptosis in the peri‐infarct region.

**Methods:**

MCAO was induced in mice using the Longa method. cTBS was administered daily for seven days. Motor function and limb coordination were assessed behaviorally. Transcriptomic and proteomic analyses of the peri‐infarct region were performed to explore mechanisms. Immunofluorescence and Western blot analyzed key proteins in the calcineurin/AKT/FOXO1 pathway, while TUNEL staining assessed neuronal apoptosis. AAV‐mediated gene knockdown was used to validate the pathway's role in apoptosis.

**Results:**

cTBS improved motor performance, reduced infarct volume, and preserved peri‐infarct tissue architecture. It suppressed calcineurin expression, enhanced calcineurin/AKT/FOXO1 pathway activation, and decreased neuronal apoptosis.

**Conclusion:**

cTBS exerts neuroprotection by modulating the calcineurin/AKT/FOXO1 pathway, ultimately inhibiting neuronal apoptosis in the peri‐infarct region.

## Graphical abstract

During the acute phase of stroke, excitotoxicity‐induced high intracellular calcium levels trigger neuronal damage, with calcineurin playing a key role in the peri‐infarct region. Continuous transcranial magnetic stimulation (cTBS) reduces calcineurin expression, activates the AKT/FOXO1 pathway, and decreases FOXO1 binding at the FasL promoter, mitigating extrinsic apoptosis and offering a novel strategy for neuroprotection after stroke.

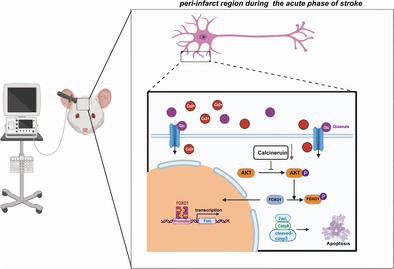

## 1 Introduction

Currently, the primary treatment for acute ischemic stroke is revascularization therapies [1, 2]. However, due to the narrow therapeutic window, only 15%–30% of patients are eligible for such treatments [3, 4, 5, 6]. Excitotoxicity is the main pathological feature during the acute phase of stroke [7, 8]. The release of neurotransmitters and alterations in neuronal excitability caused by excitotoxicity spread from the infarct core to the surrounding ischemic regions [9]. If ischemia and hypoxia in the infarct core are not effectively alleviated, neuronal death will gradually occur [10]. Studies have shown that excitotoxicity leads to neuronal apoptosis in chronic excitotoxic diseases [11, 12]. During the acute phase of stroke, apoptosis primarily appears as cell death in the peri‐infarct region, with both excitotoxicity and neuronal apoptosis occurring simultaneously. The relationship between excitotoxicity and neuronal apoptosis during the acute phase of stroke requires further investigation.

FOXO1 (Forkhead Box Protein O1) is part of the Forkhead family of transcription factors, which are controlled by various signaling pathways [13]. Notably, activation of the PI3K/AKT pathway inhibits FOXO1 function by inducing its phosphorylation at Ser256 [14, 15]. This modification enhances the nuclear export and cytoplasmic sequestration of FOXO1, ultimately decreasing its transcriptional regulatory activity. Research has shown that activating the PI3K/AKT/FOXO1 signaling pathway can inhibit ferroptosis and enhance mitochondrial autophagy [16, 17]. There is research suggesting that enhanced activation of the PI3K/AKT/FOXO1 signaling pathway can reduce cell apoptosis [18], but its effect on neurons remains unclear. However, the relationship between excitotoxicity and FOXO1 remains poorly understood.

Repetitive transcranial magnetic stimulation (rTMS) is a noninvasive neuromodulation method that uses electromagnetic induction [19, 20]. TMS has been widely used in treating depression, unipolar depression, schizophrenia, and the chronic phase of stroke [21, 22, 23, 24], but its use in the acute phase of stroke remains limited. As an inhibitory transcranial magnetic stimulation protocol, cTBS offers a potential therapeutic approach for reducing excitotoxicity and associated signaling dysregulation in acute stroke. Building on this and based on the inhibitory nature of cTBS and the role of excitotoxicity in acute stroke, the present study aims to investigate whether cTBS can reduce excitotoxicity in the peri‐infarct region, modulate the PI3K/AKT/FOXO1 signaling pathway, and ultimately decrease neuronal apoptosis in the peri‐infarct region.

## 2 Materials and Methods

### 2.1 Animals

Male C57BL/6 mice (7–9 weeks old, 21–23 g) were purchased from Zhejiang Vital River Laboratory Animal Technology Co. Ltd. (China) and housed under SPF conditions at 23°C–25°C with a 12‐h light/dark cycle. Mice had free access to a standard rodent diet. All procedures complied with NIH guidelines. MK‐2206 (MedChemExpress, HY‐10358, USA) and clozapine‐N‐oxide (BrainVTA, China) were prepared according to the manufacturers' instructions.

### 2.2 MCAO Model

The middle cerebral artery occlusion (MCAO) model of left middle cerebral artery ischemia for 60 min was produced as previously described [25]. Briefly, mice were anesthetized with intraperitoneal pentobarbital sodium (50 mg/kg), and the neck was shaved and disinfected. A midline incision exposed the CCA, ECA, and ICA under a stereomicroscope. After ligating the CCA and ECA, a silicone‐coated nylon filament was inserted via the ECA into the ICA until MCA occlusion was reached. The filament remained for 60 min, then was withdrawn to allow reperfusion. Body temperature was maintained at 37°C, and vital signs were monitored throughout.

### 2.3 Application of cTBS

cTBS was delivered using a YRD CCY‐II magnetic stimulator (Yiruide, China) with a 50 Hz triplet repeated every 200 ms. Stimulation was applied for 5 min to the infarcted hemisphere via a round coil [26]. Mice were habituated to gentle restraint for 3 days prior. Three hours post‐MCAO, awake mice received daily cTBS for 7 consecutive days, starting from day 1. During stimulation, mice were restrained with a soft glove to maintain coil proximity. For sham stimulation, the coil was positioned 15 cm above the head to mimic acoustic effects.

#### 2.3.1 Behavioral Assessment

##### 2.3.1.1 Gait Analysis

Mouse gait and posture were assessed using the CatWalk XT system (Noldus, Netherlands), which records illuminated paw prints via a high‐speed camera during locomotion on a glass walkway in the dark [27]. The system automatically analyzed key gait parameters, including run duration, average speed, stance and swing phases, step cycle, and stride length.

##### 2.3.1.2 Open Field Test (OFT)

To standardize performance, mice underwent a 1‐day acclimation with three walking trials spaced 10 min apart. Post‐MCAO locomotor activity and exploration were assessed using the Behavior Atlas system (BAYONE, China), which recorded 30‐min open‐field trajectories. Data were analyzed with the Behavior Atlas Analyzer according to the manufacturer's guidelines [28].

##### 2.3.1.3 Rotarod Test

Motor coordination and balance were assessed using a rotarod (Ugo Basile, Italy). Mice were acclimated for one day before testing. The rod accelerated from 4 to 40 rpm, and fall latency—the time until the mouse fell—was recorded. A 10‐min rest was provided between trials [25].

##### 2.3.1.4 Grid Test

Mice were placed on a metal grid (12 mm × 12 mm holes, 0.8 mm wire diameter), then inverted 40 cm above the floor after posture stabilization. Fall latency was recorded across three trials per mouse, with 5‐min intervals. The average fall time was used as the outcome measure [25].

##### 2.3.1.5 Modified Neurological Severity Score (mNSS)

Neurological function was evaluated on days 1 and 7 post‐MCAO using the mNSS, which includes tests for tail suspension, floor walking, balance, and reflexes. Scores range from 0 to 18, with 1–6 indicating mild, 7–12 moderate, and 13–18 severe injury [26].

### 2.4 Western Blotting

Cortical tissue adjacent to the infarct was lysed in RIPA buffer (Beyotime, China) with protease and phosphatase inhibitors. Nuclear and cytoplasmic proteins were extracted separately using the ExKine kit (Abbkine, China). Equal protein amounts were separated via SDS‐PAGE, transferred to methanol‐activated PVDF membranes (Merck, Germany), and blocked with 5% nonfat milk for 2 h at room temperature. Membranes were incubated overnight at 4°C with primary antibodies (Table 1), then with HRP‐conjugated secondary antibodies (Yeasen, China) for 1 h. Protein bands were visualized using ECL (Yeasen, China) and imaged with a Gel Doc XR+ system (Bio‐Rad, USA). Band intensities were quantified using ImageJ and normalized to GAPDH or β‐ACTIN (cytoplasmic) and H3 (nuclear) [29].

**TABLE 1:** Table of primary antibodies for Western blot.

PI3K	Abcam, ab180967, USA
*p*‐PI3K	CST, 17366, USA
AKT	CST, 9272, USA
*p*‐AKT	Affinity, AF0016, China
*p*‐FOXO1	CST, 84192, USA
FOXO1	CST, 2880, USA
GAPDH	Affinity, AF7021, China
β‐Actin	Affinity, AF7018, China
H3	Affinity, BF9211, China
FasL	Abcam, ab134401, USA
Cleaved‐casp3	Affinity, AF7022, China
Casp8	Affinity, AF6442, China
Calcineurin	Abcam, ab282104, USA
HRP‐conjugated goat anti‐rabbit IgG	Yeasen, China
HRP‐conjugated goat anti‐mouse IgG	Yeasen, China

### 2.5 Immunofluorescence

Brain tissue was fixed in 4% paraformaldehyde (Biosharp, China) at 4°C for 24 h, then cryoprotected in 20% and 30% sucrose (24 h each) before embedding in OCT [30]. Coronal sections (30 μm) containing peri‐infarct regions were cut using a cryostat. Sections were blocked with 10% goat serum for 1 h at room temperature, then incubated overnight at 4°C with primary antibodies against FasL, FOXO1, NeuN, and calcineurin. Fluorescent secondary antibodies (Alexa Fluor 488, 594, and 647; Invitrogen, USA) were applied for 1 h at 37°C in the dark. Imaging was performed using an Olympus FV3000 confocal microscope, and fluorescence intensity and colocalization were analyzed with ImageJ (NIH, USA).

### 2.6 Immunohistochemistry

Paraffin‐embedded brain sections were deparaffinized in xylene, rehydrated through graded ethanol, and underwent antigen retrieval in heated Tris‐EDTA buffer (pH 9.0). Endogenous peroxidase was blocked with 3% H_2_O_2_ for 30 min at room temperature. After blocking with 2.5% normal goat serum and 2% BSA, sections were incubated overnight at 4°C with primary antibodies against FasL and calcineurin. HRP‐conjugated goat anti‐rabbit IgG (CST, USA; 1:2000) was applied for 1 h at room temperature. DAB was used for visualization, followed by hematoxylin counterstaining. Positive staining cells were quantified using ImageJ (NIH, USA) with threshold analysis [31].

### 2.7 Stereotaxic Injection and Adeno‐Associated Virus (AAV) Injection

Mice were anesthetized with 5% isoflurane (maintained at 1.5%) and secured in a stereotaxic frame (RWD, China). Body temperature was maintained at 37°C with a heating pad, and eye ointment was applied to prevent corneal drying. After scalp shaving and iodine disinfection, a midline incision exposed the skull. Coordinates were calibrated using bregma and lambda [32]. A craniotomy was performed at AP‐3.0 mm, ML ±1.4 mm, DV‐1.55 mm. A glass micropipette was used to inject 0.3 μL of virus at 1 nL/S. To prevent backflow, the needle remained in place for 5 min post‐injection. Mice recovered on a heating pad and were monitored for 2 h, with daily wound checks.

### 2.8 Tissue Dissociation and Magnetic Bead Sorting

Cortical tissue was dissociated using the Neural Tissue Dissociation Kit (Miltenyi, Germany). After enzymatic digestion at 37°C for 30 min, the tissue was gently triturated and filtered through a 40 μm strainer to obtain a single‐cell suspension. Nonneuronal cells were labeled with a biotin‐antibody cocktail and removed via MACS using an LS column. Neurons were collected from the flow‐through [33].

### 2.9 chIP–qPCR

ChIP was performed using the SimpleChIP Kit (CST, USA) per the manufacturer's instructions [34]. Magnetically isolated neurons were crosslinked with 1% formaldehyde for 8 min and quenched with 0.125 M glycine. Immunoprecipitation was conducted with anti‐FOXO1 antibody (CST, #2880) or control IgG (CST, #5415). Precipitated DNA was analyzed by qPCR using SimpleChIP Universal qPCR Master Mix (CST, #88989 s). FasL promoter enrichment was assessed using primers: forward 5′‐AGCAGTCAGCGTCAGAGTTC‐3′, reverse 5′‐GTACTGGGGTTGGCTCACG‐3′.

### 2.10 Transcriptomics

Total RNA was extracted and treated with DNase to remove genomic DNA. Libraries were prepared via purification, fragmentation, end‐repair, and A‐tailing, then sequenced on an Illumina NovaSeq X Plus platform. After quality filtering, clean reads were aligned to the mouse reference genome (GRCm39). Gene expression was normalized to CPM using edgeR, and differentially expressed genes (DEGs) were identified (|fold change| ≥ 2, *p* ≤ 0.05). DEGs were subjected to GO and KEGG enrichment analyses [30].

### 2.11 Proteomics

Cortical tissue from the ischemic penumbra was collected from three cTBS‐treated MCAO mice and three untreated MCAO mice (I/R) at day 7 post‐surgery. Protein extracts were digested and fractionated via high‐pH reversed‐phase chromatography, followed by nanoLC‐MS/MS in DIA mode (MS1: 120,000; MS2: 30,000 resolution, m/z 350–1500). Spectral libraries were generated from DDA data using the Pulsar algorithm with FDR ≤ 1%. Protein quantification was based on the top three peptides per protein. Differential expression was defined as |fold change| ≥ 1.2 and *p* < 0.05 [34].

### 2.12 Apoptosis Array

Apoptosis in peri‐infarct tissue post‐MCAO/R was assessed using a semi‐quantitative protein array (RayBiotech, USA) following the manufacturer's protocol. Quality controls included biotinylated antibody spots (POS), nonspecific binding controls (NEG), and background correction spots (BLANK). Signal intensities were quantified in ImageJ by averaging duplicate spots. Normalization was performed using the formula: X(Ny) = X(y) × P(y)/P1, where X(y) is the raw signal, P(y) is the array‐specific positive control average, and P1 is the reference membrane's positive control mean [35].

### 2.13 HE And Nissl Staining

After PBS perfusion and fixation in 4% PFA, mouse brains were paraffin‐embedded and sectioned at 5 μm for H&E and Nissl staining. Nissl staining was performed with 0.1% thionine (Solarbio, China) at 37°C for 20 min following standard dehydration, clearing, and glycerol mounting procedures. Images were acquired on an Olympus VS200 microscope [25].

### 2.14 TTC Staining

On day 3 post‐MCAO/R, mice were transcardially perfused with cold PBS to clear blood, and brains were collected. Coronal brain slices (1 mm) were incubated in 2% TTC solution (Solarbio, China) at room temperature for 15 min in the dark. Viable tissue stained red, while ischemic areas lacking dehydrogenase activity appeared pale [29].

### 2.15 TUNEL Staining

Neuronal apoptosis in the peri‐infarct region was assessed using a TUNEL kit following the manufacturer's instructions. Coronal frozen sections were prepared, and neurons were identified via NeuN immunostaining. Fluorescence images were captured, and apoptotic cells were quantified by counting TUNEL‐positive cells [30].

### 2.16 Statistical Analysis

The data are presented as the means ± SDs [25]. Statistical analysis was performed via R (version 4.4.1) to evaluate the data. We employed Student's *t*‐test in conjunction with one‐way or two‐way analysis of variance (ANOVA), followed by Tukey's multiple comparisons test. A *p*‐value of less than 0.05 was regarded as statistically significant.

## 3 Results

### 3.1 cTBS Improves Health Status, Motor Function, and Exploratory Abilities in MCAO/R Mice

**FIGURE 1:**
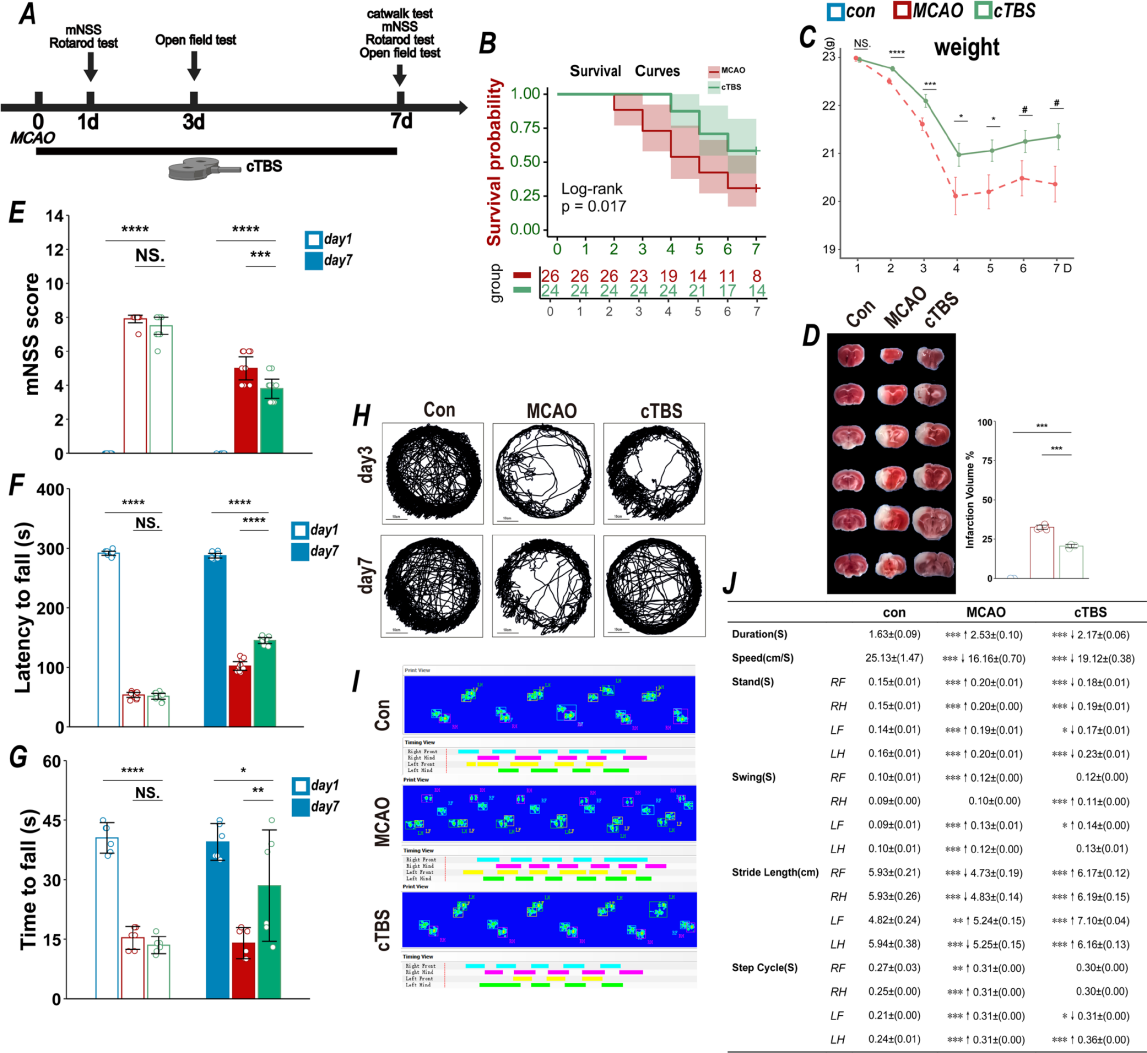
cTBS Improves Health Status, Motor Function, and Exploratory Abilities in MCAO/R Mice. (A) Timeline of experimental interventions; (B) Survival curves of the cTBS and MCAO groups; (C) Body weight changes of mice in both groups on day 7 after operation; (D) TTC staining (*n* = 6); (E) Modified neurological severity scores (*n* = 10); (F) Rotarod test (*n* = 6); (G) Grid‐walking test (*n* = 6); (H) Open field test; (I, J) CatWalk gait analysis (*n* = 5): An increase in gait score indicates functional improvement, whereas a decrease reflects impairment. 0.05 < # < 0.1, *p < 0.05, **p < 0.01, ***p < 0.001, ****p < 0.0001; NS, no significance.

The neuroprotective effects of cTBS during the acute phase of stroke were assessed in the MCAO/R model using C57BL/6 mice. As outlined in the experimental timeline, all mice underwent a series of behavioral tests: mNSS and Rotarod test on postoperative days 1 and 7, the OFT on days 3 and 7, and gait analysis on day 7, followed by the collection of brain tissue samples (Figure 1A).

To assess the changes in the health status of MCAO/R mice, weight fluctuations and survival rates were monitored. The results showed that cTBS significantly improved the survival rate of MCAO mice (Figure 1B, *p* = 0.017). Additionally, cTBS reduced stroke‐induced weight fluctuations (Figure 1C, P_D2_ < 0.0001, P_D3_ < 0.001, P_D4_ < 0.05, P_D5_ < 0.05). TTC staining further demonstrated that cTBS significantly reduced the infarct volume caused by ischemic stroke (Figure 1D,P < 0.001). Additionally, the therapeutic effects of cTBS on the recovery of neurological function in the MCAO/R mice were examined. Behavioral assessments, including the mNSS, Rotarod, and grid walking tests, revealed that cTBS treatment significantly improved motor function, muscle strength, and balance ability (Figure 1E–G; P_mNSS_ < 0.001, P_rotarod_ < 0.0001, P_grid_ < 0.01). The results of OFT on days 3 and 7 after MCAO surgery demonstrated that the cTBS group exhibited denser movement trajectories, indicating stronger motor and exploratory abilities at both time points (Figure 1H). Gait analysis revealed that the cTBS group exhibited significant improvements in motor function and coordination of the contralateral limb (Figure 1I,J). These findings collectively support the neuroprotective role of cTBS in MCAO/R mice.

### 3.2 Transcriptomic and Proteomic Analyses Suggest That cTBS May Exert Neuroprotective Effects Through Calcium Signaling Pathway and Apoptosis

**FIGURE 2:**
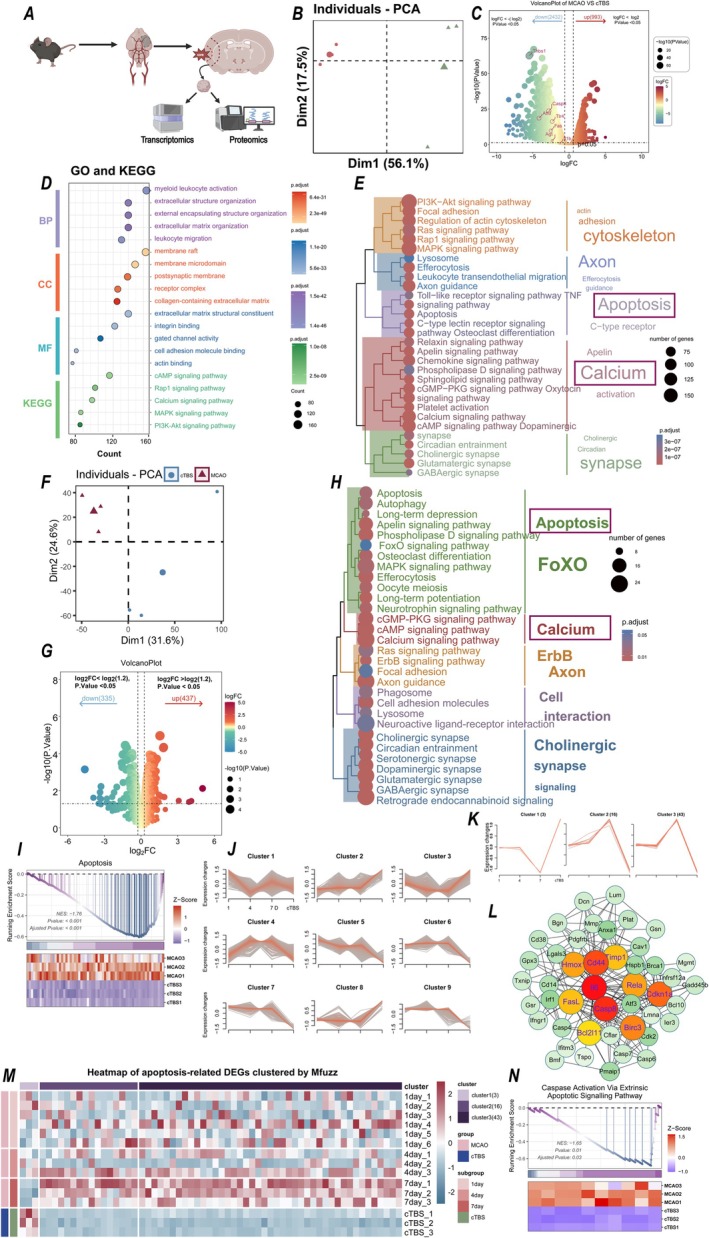
Transcriptomic and proteomic analyses of the peri‐infarct region in mice subjected to cTBS vs. MCAO. (A) Schematic representation of sample collection for transcriptomic and proteomic analyses (*n* = 3); (B) PCA of transcriptomic samples; (C) Volcano plot of DEGs between the two transcriptomic groups, with thresholds set at |FC| ≥ 2 and *p* ≤ 0.05; (D) *Key* outcomes of enrichment analyses for GO and the KEGG; (E) De‐redundancy and visualization of all KEGG enrichment results from the transcriptomics; (F) PCA of proteomic samples; (G) Volcano plot of DEPs between the two proteomic groups, with thresholds set at |FC| ≥ 1.2 and *p* ≤ 0.05; (H) De‐redundancy analysis and visualization of all GO enrichment results derived from proteomic data; (I) Gene set enrichment analysis (GSEA) of the transcriptome showing suppression of apoptosis; (J) Clustering results of time‐series analysis integrating GEO datasets (GSE213177 and GSE231718) with transcriptomic data; (K) Expression patterns of selected time‐clustered genes associated with apoptosis; (L) Protein–protein interaction (PPI) network of apoptosis‐related genes at the proteomic level; (M) Heatmap illustrating the expression profiles of apoptosis‐related genes; (N) GSEA of the Caspase Activation via extrinsic apoptosis signaling pathway.

To further investigate the potential mechanisms underlying the neurofunctional recovery induced by cTBS in MCAO/R mice, transcriptomic and proteomic analyses were performed on the peri‐infarct region of both the MCAO and cTBS groups (Figure 2A). Transcriptomic analysis identified 993 upregulated and 2432 downregulated genes in the peri‐infarct region (|fold change| > 2, *p* < 0.05, Figure 2B,C). The GO and KEGG enrichment analysis results of DEGs were further clustered (Figure 2D), focusing on the key biological processes influenced by cTBS. The results suggest that the effects of cTBS in the peri‐infarct region might involve the modulation of processes such as morphogenesis, intracellular activity, signal transduction, calcium signaling, and apoptosis (Figure 2E, Figure S1A).

Proteomic analysis identified 437 upregulated proteins and 335 downregulated proteins in the peri‐infarct region (Figure 2F,G). Clustering of the GO and KEGG enrichment results for DEPs revealed that synaptic activity, apoptosis, calcium signaling, and the FOXO signaling pathway were central to the mechanisms through which cTBS exerts its effects in the peri‐infarct region (Figure 2H, Figure S1B,C). Both the transcriptomic and proteomic analyses highlight the significance of calcium signaling and neuronal apoptosis as key processes in the action of cTBS. Furthermore, GSEA of the total gene showed that apoptosis was significantly inhibited in the peri‐infarct region (Figure 2I).

To clarify the molecular mechanisms by which cTBS inhibits apoptosis, transcriptomic data were integrated with GEO datasets (GSE213177 and GSE231718) to perform clustering analysis and examine gene expression patterns (Figure 2J,K). Expression patterns of apoptosis‐related differentially expressed genes were extracted, and key targets were identified through Protein–Protein Interaction (PPI) network analysis. The PPI analysis highlighted Casp8 and FasL as central nodes (Figure 2L,M), both of which are critical components of the extrinsic apoptotic pathway. Furthermore, GSEA of the DEGs indicated significant inhibition of caspase activation within this pathway (Figure 2N), suggesting that cTBS may mitigate neuronal loss by suppressing this specific apoptotic process in the peri‐infarct region.

### 3.3 cTBS Preserves Post‐Ischemic Brain Tissue Structure and Reduces the Expression of FasL‐Associated Apoptotic Proteins

**FIGURE 3:**
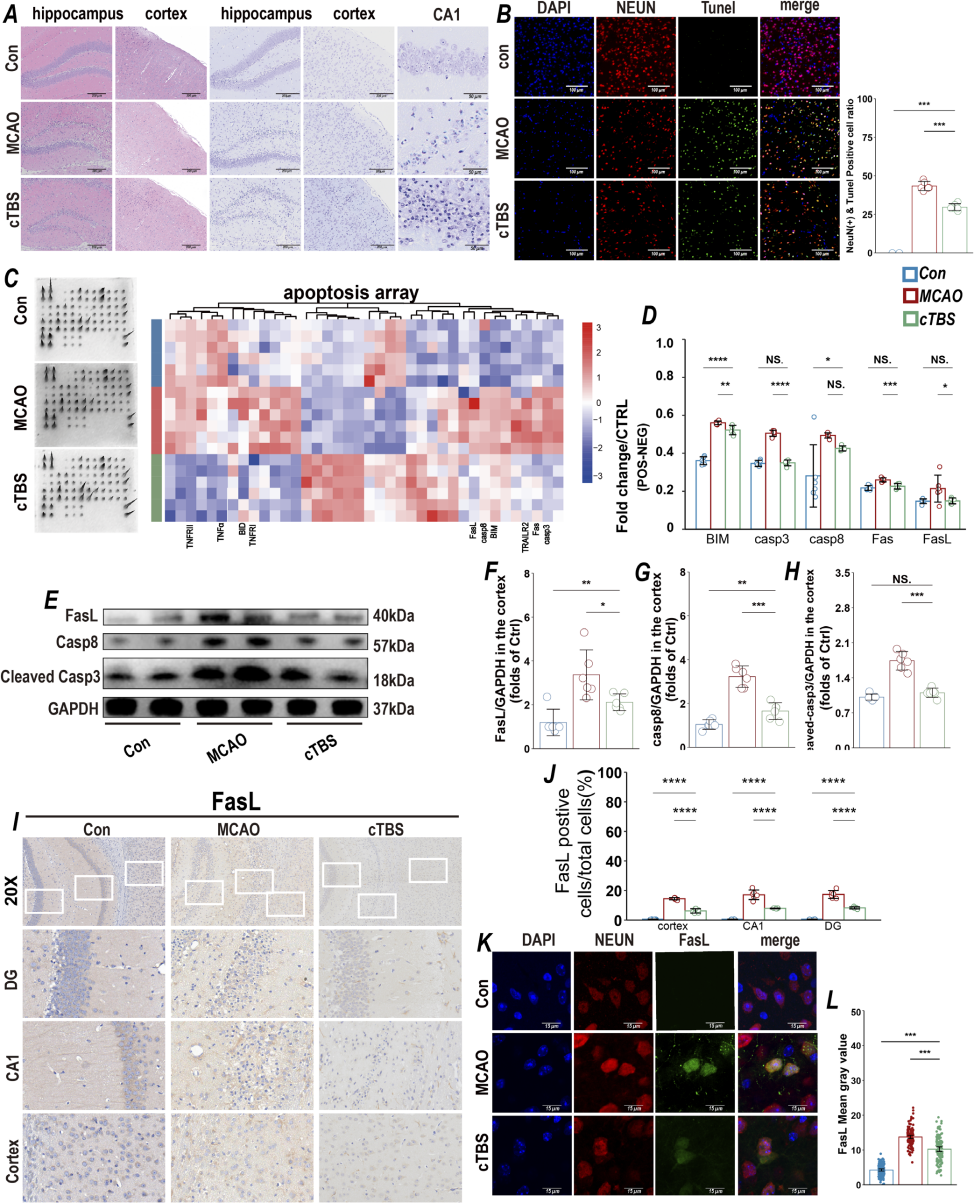
cTBS improves neuronal survival in the peri‐infarct region by inhibiting extrinsic apoptosis. (A) HE staining and Nissl staining; (B) TUNEL staining of neurons in the peri‐infarct region (*n* = 6); (C, D) Alterations in apoptotic protein levels in peri‐infarct tissue; (E–H) Expression levels of extrinsic apoptotic proteins in the peri‐infarct region (*n* = 6); (I, J) expression and distribution of FasL in the DG, CA1 region, and cortex of the peri‐infarct region(*n* = 6); (K, L) Representative immunofluorescence images of NeuN and FasL co‐staining, scale bar = 15 μm (*n* = 100–120 cells per group). *p < 0.05, **p < 0.01, ***p < 0.001, ****p < 0.0001; NS, no significance.

The morphological effects of cTBS were first assessed using HE and Nissl staining. The HE staining results showed that cTBS improved stroke‐induced morphological damage. Nissl staining revealed that cTBS treatment increased the number of Nissl bodies in neurons of the hippocampus, cortex, and CA1 region (Figure 3A). Additionally, TUNEL staining further confirmed that cTBS significantly decreased the number of apoptotic neurons in the peri‐infarct region, supporting its neuroprotective effects (Figure 3B, P_TUNEL_ < 0.001).

The expression levels of apoptosis‐related factors were next assessed using an apoptosis array in the peri‐infarct region. The result showed that cTBS significantly inhibited the expression of extrinsic apoptotic factors, including BIM, caspase‐3, FasL, and Fas (Figure 3C,D; P_BIM_ < 0.01, P_casp3_ < 0.0001, P_Fas_ < 0.001, P_FasL_ < 0.05). Transcriptomic and apoptosis array results collectively revealed that cTBS inhibited the expression of extrinsic apoptotic factors. Furthermore, Western blotting results further confirmed that cTBS significantly suppressed the expression of FasL, caspase‐8, and cleaved caspase‐3 in the contralateral infarct region (Figure 3E–H; P_FasL_ < 0.05, P_casp8_ < 0.001, P_casp3_ < 0.001).

Immunohistochemistry results showed that cTBS significantly reduced the number of FasL‐positive cells in the cortex, CA1, and DG regions (Figure 3I,J; P_cortex_ < 0.001, PC_A1_ < 0.001, P_DG_ < 0.001). Furthermore, immunofluorescence results indicated that cTBS inhibited the expression level of FasL within neurons in the peri‐infarct region(Figure 3K,L, *p* < 0.001). Taken together, these results indicate that cTBS exerts its neuroprotective effects by suppressing FasL expression, thereby reducing neuronal apoptosis in the peri‐infarct region.

### 3.4 cTBS Attenuates the Expression of FasL‐Associated Apoptotic Proteins Through Activation of the AKT/FOXO1 Signaling Pathway

Recent studies have shown that FasL is a target gene of FOXO1 [15, 36, 37]. Based on the transcriptomic results, the upstream pathway of FasL was identified as the PI3K/AKT/FOXO1 signaling pathway. We hypothesize that cTBS may exert its neuroprotective effects by modulating the phosphorylation status of FOXO1 through the PI3K/AKT signaling pathway, thereby inhibiting extrinsic apoptosis.

**FIGURE 4:**
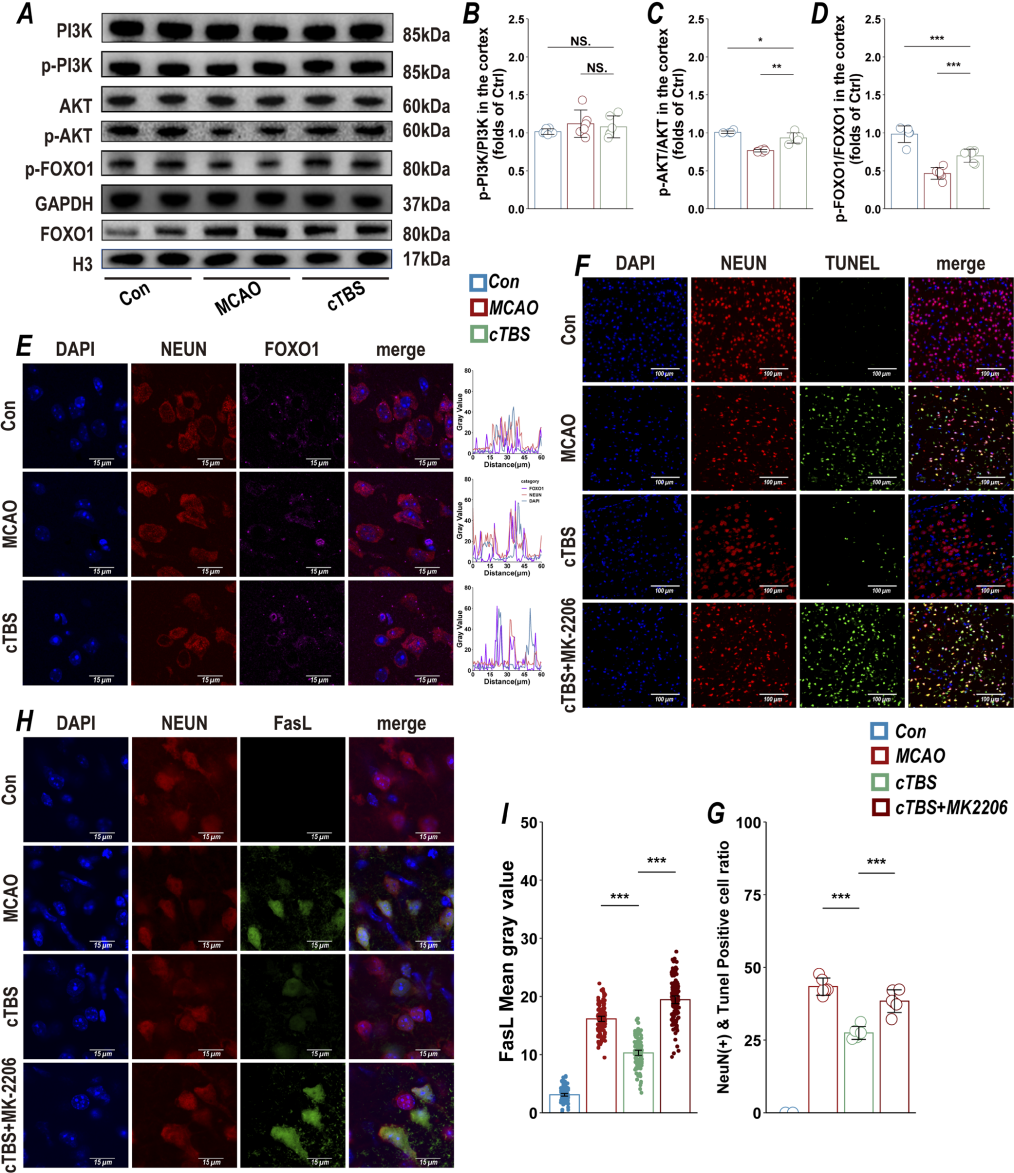
cTBS inhibits extrinsic apoptosis in the peri‐infarct region through the AKT/FOXO1 signaling pathway. (A–D) Protein expression levels of the PI3K/Akt/FOXO1 signaling pathway in the peri‐infarct region; (E) Representative immunofluorescence images showing NeuN and FOXO1 co‐staining, scale bar = 15 μm; (F, G) TUNEL staining of neurons in the peri‐infarct region (*n* = 6), scale bar = 100 μm; (H, I) Representative immunofluorescence images of NeuN and FasL co‐staining, scale bar = 15 μm (*n* = 100–120 cells per group); *p < 0.05, **p < 0.01, ***p < 0.001, ****p < 0.0001; NS, no significance.

To test this hypothesis, the expression levels of key proteins in the PI3K/AKT/FOXO1 signaling pathway were first assessed using Western blotting. The results revealed that cTBS significantly increased the ratio of p‐AKT/AKT and p‐FOXO1/FOXO1 (Figure 4A–D; P_p‐PI3K/PI3K_ > 0.05, P_p‐AKT/AKT_ < 0.01, P_p‐FOXO1/FOXO1_ < 0.001). Immunofluorescence analysis further demonstrated that cTBS promoted the translocation of FOXO1 from the nucleus to the cytoplasm (Figure 4E), suggesting that cTBS may promote FOXO1 phosphorylation and inhibit extrinsic apoptosis in neurons in the peri‐infarct region.

To further investigate whether cTBS regulates FOXO1 phosphorylation via AKT activation and thereby reduces neuronal apoptosis in the peri‐infarct region, we conducted additional experiments in MCAO/R mice using the AKT inhibitor MK2206. TUNEL staining results showed that inhibition of the AKT/FOXO1 signaling pathway reversed the neuroprotective effects of cTBS (Figure 4F,G; P_TUNEL_ < 0.001). Furthermore, Immunofluorescence analysis further supported that cTBS inhibits neuronal FasL expression levels through the AKT/FOXO1 signaling pathway (Figure 4H,I; P_IF_ < 0.001). Taken together, these findings suggest that cTBS exerts its neuroprotective effects by modulating the AKT/FOXO1 signaling pathway, which in turn inhibits FasL expression and reduces neuronal apoptosis in the peri‐infarct region.

### 3.5 During the Acute Phase of Ischemic Stroke, cTBS Activates the AKT/FOXO1 Signaling Pathway by Inhibiting the Calcium Signaling Pathway

**FIGURE 5:**
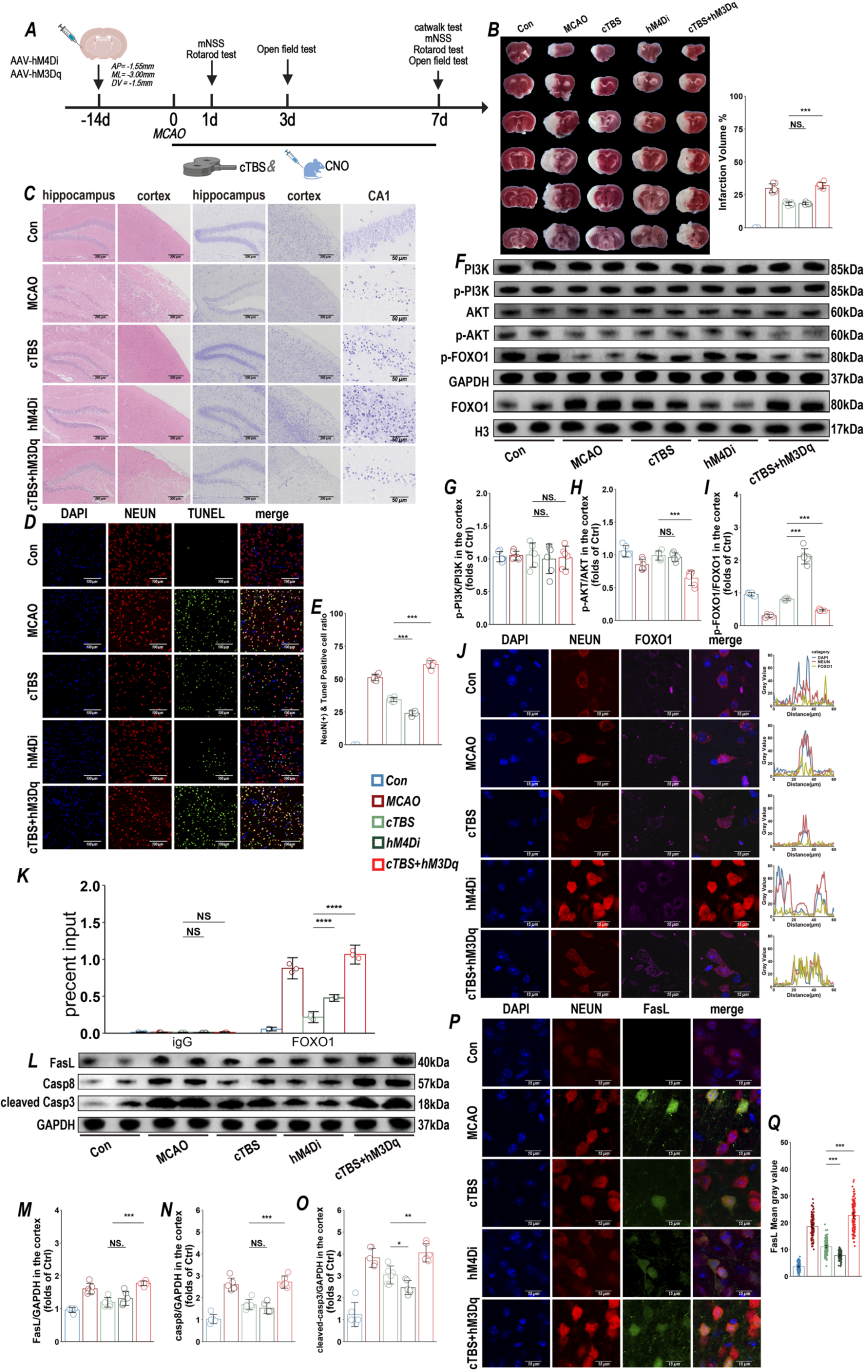
cTBS inhibits neuronal apoptosis in the peri‐infarct region through modulation of calcium signaling pathway activity and phosphorylation of FOXO1. (A) Timeline of experimental interventions; (B) TTC staining (*n* = 6); (C) HE staining and NISSL staining; (D, E) TTC staining (*n* = 6); (F–J) Expression changes in the PI3K/Akt/FOXO1 signaling pathway in the peri‐infarct region (*n* = 6); (K) Representative immunofluorescence images of NeuN and FOXO1 co‐staining, scale bar = 15 μm; (L) Enrichment of the FOXO1 transcription factor at the FasL gene promoter in neurons of the peri‐infarct region (*n* = 3); (M–P) Expression levels of extrinsic apoptotic proteins (*n* = 6); (Q) Representative immunofluorescence images of NeuN and FasL co‐staining, scale bar = 15 μm (*n* = 100–120 cells per group). *p < 0.05, **p < 0.01, ***p < 0.001, ****p < 0.0001; NS, no significance.

Transcriptomic and proteomic analyses suggest that the calcium signaling pathway plays a key role in cTBS‐mediated neuroprotection. To investigate its involvement in modulating the PI3K/AKT/FOXO1 pathway, we injected AAV‐hM4Di (to inhibit calcium signaling) or AAV‐hM3Dq (to enhance calcium signaling) viruses into the cortical region supplied by the MCA in mice. The experimental groups were: con, MCAO, cTBS, hM4Di, and cTBS + hM3Dq (Figure 5A).

TTC staining showed that cTBS and inhibition of calcium signaling similarly reduced infarct volume, whereas enhancing calcium signaling reversed the therapeutic effects of cTBS (Figure 5B, P_TTC_ < 0.001). HE and Nissl staining further revealed that enhanced calcium signaling during the acute phase of ischemic stroke reversed the tissue protective effects of cTBS in the peri‐infarct region (Figure 5C). TUNEL staining also indicated that enhanced calcium signaling reversed the inhibitory effects of cTBS on neuronal apoptosis (Figure 5D,E; P_TUNEL_ < 0.001).

To explore the relationship between calcium signaling and the PI3K/AKT pathway, the phosphorylation levels of key proteins were first assessed. Western blotting showed that inhibiting calcium signaling increased the p‐FOXO1/FOXO1 ratio (*p* < 0.001), whereas enhancing calcium signaling combined with cTBS significantly decreased the p‐AKT/AKT and p‐FOXO1/FOXO1 ratios (Figure 5F–I; P_p‐AKT/AKT_ < 0.001, P_p‐FOXO1/FOXO1_ < 0.001). Notably, no significant difference was observed in the p‐PI3K/PI3K ratio, suggesting that calcium signaling regulates FOXO1 phosphorylation independently of PI3K activation (Figure 5G, P_p‐PI3K/PI3K_ > 0.05). Immunofluorescence and ChIP‐qPCR results collectively confirmed that the effect of cTBS on the AKT/FOXO1 signaling pathway was blocked by enhanced calcium signaling (Figure 5J,K, P_ChIP‐qPCR_ < 0.0001). We assessed the expression levels of downstream proteins of the AKT/FOXO1 pathway. The results of Western blotting and immunofluorescence showed that the inhibitory effect of cTBS on FasL/casp8/casp3 was abolished upon enhancement of Ca signaling (Figure 5L–Q, P_FasL_ < 0.001, P_Casp8_ < 0.001, P_Cleaved‐casp3_ < 0.01).

In summary, these findings collectively demonstrate that cTBS may exert its neuroprotective effects by regulating the activity of the AKT/FOXO1 signaling pathway through modulation of the calcium signaling pathway.

### 3.6 cTBS Exerts Neuroprotective Effects by Activating the Calcineurin/AKT/FOXO1 Signaling Pathway, Thereby Inhibiting Neuronal Apoptosis

**FIGURE 6:**
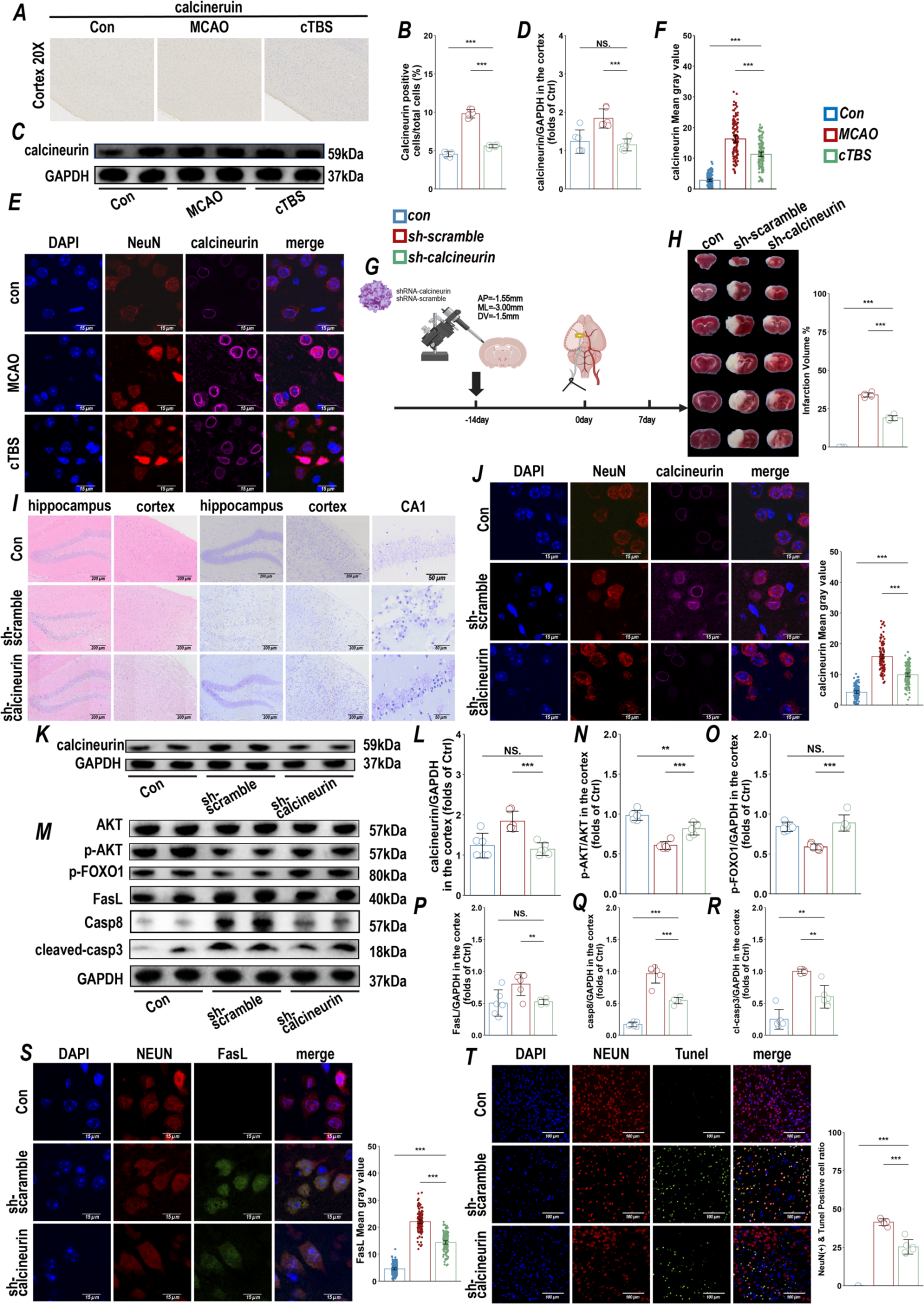
cTBS exerts neuroprotective effects by inhibiting extrinsic apoptosis through the calcineurin/AKT/FOXO1 signaling pathway. (A, B) Immunohistochemical staining of calcineurin in the peri‐infarct region (*n* = 6); (C, D) Expression levels of the Ca^2+^‐associated protein calcineurin (*n* = 6); (E, F) Immunohistochemical staining of calcineurin in the peri‐infarct region (*n* = 6); (G) Experimental timeline for calcineurin knockdown in neurons of the peri‐infarct region; (H) TTC staining (*n* = 6); (I) HE staining and NISSL staining; (J) Representative immunofluorescence images showing NeuN and calcineurin co‐staining, scale bar = 15 μm; (K, L) Expression levels of the Ca^2+^‐associated protein calcineurin (*n* = 6); (M–R) Protein expression levels of the Akt/FOXO1 signaling pathway (*n* = 6); (S) Representative immunofluorescence images of NeuN and FasL co‐staining, scale bar = 15 μm (*n* = 100–120 cells per group); (T) TUNEL staining of neurons in the peri‐infarct region, scale bar = 100 μm. *p < 0.05, **p < 0.01, ***p < 0.001, ****p < 0.0001; NS, no significance.

Recent studies indicate that calcineurin, a calcium‐dependent protein, is regulated by intracellular calcium concentrations. Calcineurin plays critical roles in various biological processes, including the antagonism of AKT activity by inhibiting AKT phosphorylation and its downstream signaling cascades [38]. Recent studies show that the persistent activation of calcineurin in Alzheimer's disease results in the downregulation of genes related to synapses, which then leads to cognitive impairment [39]. The GSEA of the transcriptome shows that cTBS suppresses calcium‐mediated signaling in the peri‐infarct area (Figure S1D). We hypothesize that the expression level of calcineurin is elevated in the peri‐infarct region during the acute phase of stroke. The results of Western blotting, immunohistochemistry, and immunofluorescence collectively revealed that the expression of calcineurin was significantly higher in the peri‐infarct area, and that cTBS could inhibit the expression of calcineurin (Figure 6A–F; P_IHC_ < 0.001, P_WB_ < 0.001, P_IF_ < 0.001).

To further elucidate the regulatory interplay between calcineurin and the PI3K/AKT signaling pathway, we specifically modulated calcineurin expression in neurons of the peri‐infarct region. The experimental groups included con, sh‐scramble, and sh‐calcineurin groups (Figure 6G). The results of TTC staining, HE staining, and Nissl staining collectively indicated that knockdown of neuronal calcineurin significantly attenuated the infarct volume and preserved the structural integrity of the tissue. (Figure 6H,I; P_TTC_ < 0.001). Both immunofluorescence and Western blotting results validated the efficiency of AAV‐mediated knockdown (Figure 6J–L; P_IF_ < 0.001, P_WB_ < 0.001).

Western blotting and immunofluorescence analysis revealed that knockdown of neuronal calcineurin resulted in a significant decrease in the expression levels of the extrinsic apoptotic markers FasL, casp8, and cleaved‐casp3, indicating a robust activation of the AKT/FOXO1 signaling pathway during the acute phase of stroke (Figure 6M–S; P_p‐AKT/AKT_ < 0.001, P_p‐FOXO1/FOXO1_ < 0.001, P_FasL_ < 0.01, P_casp8_ < 0.01, P_casp3_ < 0.01, P_IF_ < 0.01). TUNEL staining further demonstrated that calcineurin knockdown significantly reduced neuronal apoptosis (Figure 6T, P_TUNEL_ < 0.001). These findings suggest that inhibiting calcineurin expression in neurons can activate the AKT/FOXO1 signaling pathway, thereby suppressing extrinsic apoptosis and providing neuroprotection. Overall, cTBS exerts its neuroprotective effects by inhibiting calcineurin expression, activating the AKT/FOXO1 signaling pathway, and suppressing neuronal apoptosis in the peri‐infarct region during the acute phase of stroke.

## 4 Discussion

cTBS inhibits neuronal excitability, providing a rationale for its application to the affected hemisphere during the acute phase of ischemic stroke [20]. Previous studies have shown that cTBS suppresses neuronal apoptosis in the peri‐infarct region, consistent with our findings. While prior work primarily addressed its effects on neuroinflammation, such as microglia and astrocyte subtypes, our study focuses on the direct impact of cTBS on peri‐infarct neurons to elucidate its underlying mechanisms.

Behavioral analyses demonstrated that cTBS improves motor function, coordination, and exploratory behavior in mice during the acute phase of ischemia. Integrated transcriptomic and proteomic analyses, including KEGG and GO enrichment, highlighted calcium signaling and apoptosis as key pathways mediating cTBS neuroprotection. GSEA further confirmed that cTBS suppresses apoptosis in peri‐infarct neurons, supporting apoptosis as the most downstream event of its protective effect. Temporal profiling revealed that apoptosis‐related genes progressively increase over time, whereas 7‐day cTBS treatment significantly attenuated this trend. PPI analysis suggested that cTBS may regulate apoptosis through FasL.

FasL, a key mediator of extrinsic apoptosis, cooperates with caspase‐8 and cleaved caspase‐3 to initiate the apoptotic cascade [36, 37]. GSEA indicated that cTBS inhibits caspase activation in peri‐infarct neurons, and experiments confirmed that cTBS markedly reduces neuronal FasL expression. Mechanistically, the PI3K/AKT/FOXO1 pathway was identified as an upstream regulator, and pharmacological inhibition of AKT with MK‐2206 validated that cTBS modulates FasL expression and apoptosis via this axis. Together, these data indicate that cTBS suppresses neuronal apoptosis in the peri‐infarct region by regulating FasL through AKT/FOXO1 signaling.

Transcriptomic analyses also implicated calcium signaling as a critical upstream mediator. Although its role in apoptosis is not fully defined, GSEA suggested that cTBS suppresses calcium‐dependent signaling. Using AAV‐mediated manipulations of calcium activity in peri‐infarct neurons, we performed bidirectional validation. Western blotting showed changes in the PI3K/AKT/FOXO1 pathway and FasL/caspase‐8/cleaved‐caspase‐3, while ChIP‐qPCR confirmed FOXO1 binding to the FasL promoter. Enhancing neuronal calcium activity counteracted cTBS effects on the AKT/FOXO1 pathway, indicating that calcium signaling lies upstream. Literature reports that the calcium‐dependent protein calcineurin modulates AKT phosphorylation [38]; accordingly, cTBS suppressed calcineurin expression, consistent with its inhibition of calcium‐mediated activity. Moreover, AAV‐mediated knockdown of calcineurin in peri‐infarct neurons reduced infarct volume and apoptosis, increased the p‐AKT/AKT ratio, and decreased FasL expression. In summary, our study demonstrates that cTBS exerts neuroprotective effects during the acute phase of cerebral ischemia. The underlying mechanism appears to involve the downregulation of calcineurin, a key protein in the calcium signaling pathway, thereby promoting the Akt/FOXO1 signaling pathway.

This study demonstrates the neuroprotective effects of cTBS during the acute phase of ischemic stroke. Its non‐invasive nature makes cTBS a promising therapeutic strategy with broad clinical potential, offering a novel avenue for stroke intervention. Nonetheless, several limitations should be noted. First, although transcriptomic analyses indicate that calcium signaling is a key mediator of cTBS effects, we did not directly assess intracellular calcium dynamics in real time. Future studies should employ techniques such as electrophysiological recordings or calcium‐sensitive fluorescent probes to directly evaluate the impact of cTBS on calcium signaling, thereby providing more definitive mechanistic evidence. Moreover, it is possible that cTBS exerts neuroprotection through additional, yet unidentified pathways, which warrants further investigation. Recent studies have indicated that cTBS can promote stroke recovery through multiple pathways, including vascular protection, neurovascular regeneration, mitochondrial integrity, and modulation of the neuromicroenvironment [26, 40, 41]. Furthermore, our study did not comprehensively explore the effects of cTBS on glial cells, particularly activated glial cells, and their interactions with neurons. A recent study has indicated that TMS can alleviate neuronal damage in the peri‐infarct region by promoting the polarization of M2 microglia [25]. Future research should address these factors to achieve a more comprehensive understanding of the neuroprotective mechanisms of cTBS. The role of calcineurin in neurological diseases remains inadequately explored, particularly in the context of acute ischemic stroke and other excitotoxicity‐related conditions. Given its involvement in neuronal apoptosis, calcineurin may represent a potential therapeutic target. Our study confirms that cTBS improves neuronal apoptosis by inhibiting the expression of calcineurin. We hope that this research provides a foundation for the clinical use of cTBS in the acute phase of stroke.

## Funding

This study was supported by this research was supported by the National Natural Science Foundation of China (Grant Nos. 82571927, 82302865, 82202799, 82172544, and 82072547), the National Key Research and Development Program of China (Grant No. 2023YFC3604801), and the Shanghai Science and Technology Committee Sailing Program (Grant No. 23YF1403800).

## Ethics Statement

All animal experiments were conducted in compliance with international ethical guidelines and approved by the Animal Ethics Committee of Fudan University (approval no. 2020‐huashan hospital‐JS‐163).

## Conflicts of Interest

The authors declare no conflicts of interest.

## Supporting information


**Figure S1:** (A) Gene Ontology (GO) enrichment analysis of the transcriptome with redundancy removal and visualization; (B) Kyoto Encyclopedia of Genes and Genomes (KEGG) enrichment analysis of the proteomics with redundancy removal and visualization; (C) Alterations in pathway‐related proteins identified by proteomic analysis; (D) Gene set enrichment analysis (GSEA) of the transcriptome showing suppression of calcium‐mediated signaling.

## Data Availability

The data that support the findings of this study are available from the corresponding author upon reasonable request.
