# Supplementary figures and images for: The Protective Mechanism of Continuous Theta Burst Stimulation in the Acute Phase of Stroke Through Modulation of the Calcineurin/AKT/FOXO1 Signaling Pathway
Source: CNS Neurosci Ther. 2026 Jul 8;32(7):e71017. doi: 10.1002/cns.71017 (PMC13343732; doi:10.1002/cns.71017)

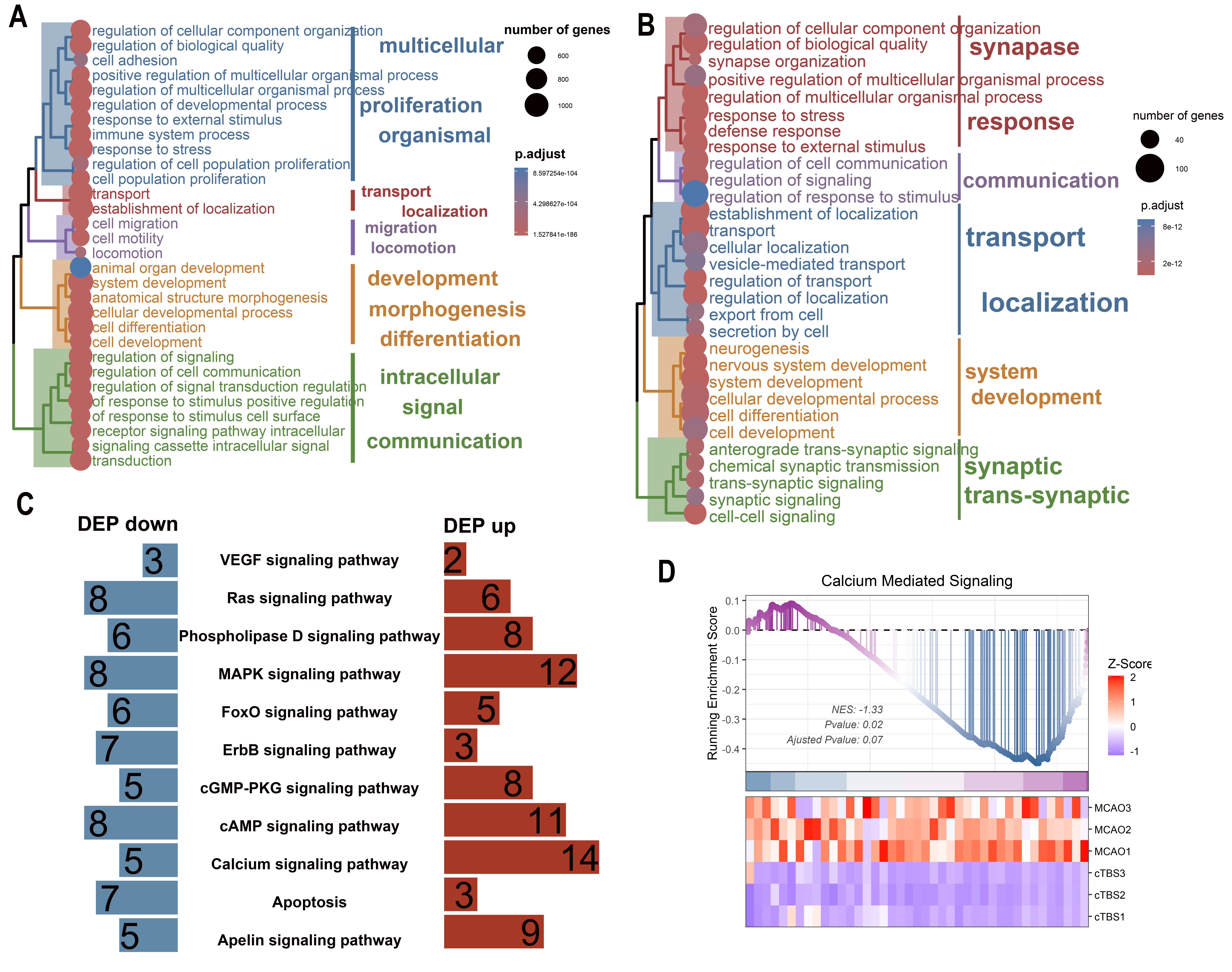

Supplement: Supplementary file 1 — Figure S1: (A) Gene Ontology (GO) enrichment analysis of the transcriptome with redundancy removal and visualization; (B) Kyoto Encyclopedia of Genes and Genomes (KEGG) enrichment analysis of the proteomics with redundancy removal and visualization; (C) Alterations in pathway‐related proteins identified by proteomic analysis; (D) Gene set enrichment analysis (GSEA) of the transcriptome showing suppression of calcium‐mediated signaling. [file CNS-32-e71017-s001.png]
